# A novel Danshensu/tetramethylpyrazine protects against Myocardial Ischemia Reperfusion Injury in rats

**DOI:** 10.7150/ijms.59411

**Published:** 2021-05-13

**Authors:** Jinghao Wang, Kai Fan, Cong He, Qingyang Wang, Qianhui Zhang, Wei Huang

**Affiliations:** 1 Department of Pharmacology, Harbin Medical University-Daqing, Daqing, 163319, China.; 2 Department of Pharmacy, the First Affiliated Hospital, Jinan University, Guangzhou 510630, China.; 3 Department of Pathophysiology, Harbin Medical University-Daqing, Daqing, 163319, China.

**Keywords:** ADTM, ischemia reperfusion, PAFR, apoptosis, inflammation

## Abstract

A new Danshensu/tetramethylpyrazine derivative (ADTM) with cardio-protection effects such as antioxidant, arterial relaxation, pro-angiogenesis and antiplatelet activities. Platelet activating factor receptor (PAFR) plays a key role in myocardial ischemia reperfusion (MIR) injury. This study aims to investigate the protective role of ADTM in MIR injury and clarify the potential role of PAFR. We measured the effects of ADTM on MIR injury in rats *in vivo* and hypoxia re-oxygenation (HR) injury in neonatal rat ventricular myocytes (NRVMs) *in vitro*. The results show that ADTM can significantly improve the IR-induced decline in heart function as increasing EF and FS, and restore the decreased cardiac hemodynamic parameters (LVSP, ± dp/dt max) and increased the level of LVEDP, decrease the infarct size of damaged myocardium and lactate dehydrogenase (LDH) activity in serum. Additionally, ADTM inhibits cardiomyocytes apoptosis, caspase-3 activity, and inflammatory response as well as down-regulates the MIR-induced IL-1β and TNFα production. Next, PAFR expression was significantly down-regulated in cardiomyocytes of MIR model *in vivo* and *in vitro* after treated with ADTM compare to IR group. At the same time, ADTM and PAFR small interfering RNA (siRNA) could inhibit cardiomyocytes apoptosis and inflammation during HR, while PAF presents the opposite effect. Furthermore, the above effects of PAF in HR induced cardiomyocytes were reversed by co-treatment of ADTM. Our findings demonstrate for the first time that ADTM protects against MIR injury through inhibition of PAFR signaling, which provides a new treatment for MIR.

## Introduction

Coronary heart disease (CHD) is a widespread public health problem with high morbidity and mortality 1. Myocardial ischemia reperfusion (MIR) injury is a main clinical outcome of CHD which is characterized by an initial limitation in blood supply, followed by restoration of perfusion and concomitant re-oxygenation, and ultimately leads to acute myocardial infarction, severe arrhythmias, or heart failure 2, 3. The main pathogenesis of MIR is related to the massive release of free oxygen radicals, loss of intracellular and mitochondrial calcium homeostasis, inflammation cascades, promotion of apoptosis and myocardial necrosis 4. Therefore, to fully understand the mechanism of MIR damage and to find new effective drugs is still the focus of research.

Apoptosis and inflammation have been considered as two main signs of MIR 5, 6. Recent evidence suggests that apoptosis is mainly triggered or accelerated during IR, and partly results in overall cardiomyocytes death 6, 7. Inhibition or blocking of the apoptosis process can prevent the loss of contractile cells, minimize IR-induced heart damage, and delay the occurrence of myocardial shock and heart failure 8. Similarly, during the entire process of MIR, a strong inflammatory response will be stimulated as it accelerates the production of inflammatory factors and simultaneously promotes inflammatory bursts 9, 10. The obstruction of inflammatory stimuli is associated with poor prognosis and increased severity of MIR, and may severely delay recovery procedures 11. Therefore, reducing the inflammatory response and apoptosis during reperfusion after ischemic injury is an advantageous therapeutic strategy against MIR diseases.

**Figure 1:**
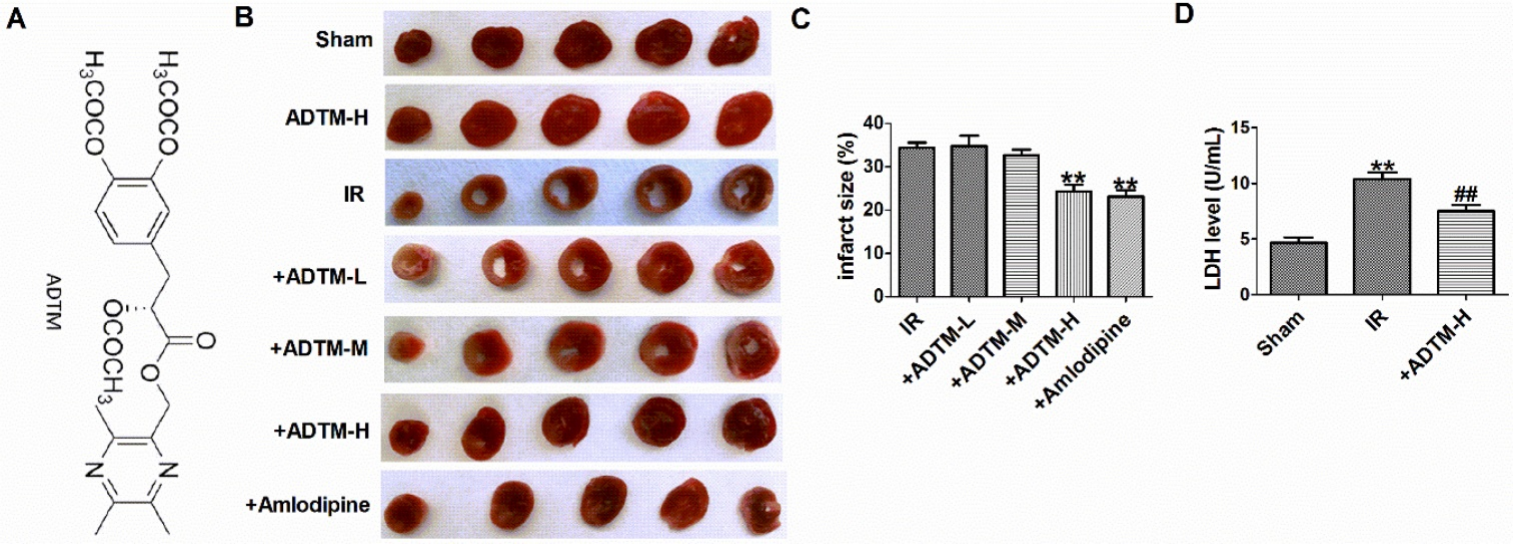
**The protective effect of ADTM on rat MIR injury.** (A) Chemical structure of ADTM. (B) Representative images showing infarct areas in cross section slices. (C) Statistical analysis of IA/LV ratio. IA: infarct area, LV: left ventricles. (D) Serum LDH activity. **P < 0.01 versus Sham; ^#^P < 0.05, ^##^P < 0.01 versus IR. n = 6.

Natural products and their derivatives have played an important role in the discovery and development of new drugs to treat MIR 12-15. Shenlian extract could protect against MIR through the NF-kB signaling pathway 12. Luteolin can attenuate MIR by activating peroxiredoxin II 13. Panaxatriol derivatives attenuated MIR injury in the rat 14. Caffeoylquinic acid derivative of Erigeron multiradiatus reduces MIR damage by inhibiting the activation of NF-kB and JNK 15. A novel type of semi-synthetic small molecule named (R)-(3,5,6-Trimethylpyrazinyl) methyl-2-acetoxy-3-(3,4-diacetoxyphenyl) propanoate (ADTM, Fig.1A), in which Danshensu and Tetramethylpyrazine are coupled via an ester bond 16, 17. ADTM has protective effects on acute myocardial infarction in animals and oxidative stress-induced cardiomyocytes injury in H9c2 cells 18-20. However, the role of ADTM in the regulating MIR and its underlying molecular mechanism remains to be studied.

In the current study, we used the rat MIR model and hypoxia/re-oxygenation -induced cardiomyocytes injury model to investigate whether ADTM has a protective effect on MIR-induced cardiomyocytes apoptosis and inflammation. Our findings indicate that ADTM may provide a new treatment for ischemic heart disease.

## Materials and methods

### Animal experiments

In this study, male Sprague-Dawley rats (220 ± 20 g) were purchased from Harbin Medical University (Harbin, China). The experimental procedures involving animals in this study were approved by the Animal Ethics Committee of Harbin Medical University and the study was carried out in accordance with the Guide for the Care and Use of Laboratory Animals published by the US National Institutes of Health (NIH Publication, 8th Edition, 2011). As mentioned previously 21, the MIR model was established by ligating the anterior descending branch of the left coronary artery. Rats were anaesthetized with ketamine-xylazine (100 mg/kg, 5 mg/kg, i.p.). Sham-operated animals undergo the same procedure, but coronary artery ligation is not tied. The electrocardiogram is recorded before and after ligation to confirm ischemia. The rats were randomly divided into the following seven experimental groups (six rats in each group): Sham operation (Sham) group, Sham group treated high dosage ADTM (24 mg/kg), ischemia reperfusion (IR) group, IR treated positive control Amlodipine (2 mg/kg) group, IR treated low dosage ADTM (6 mg/kg) group (ADTM-L, low dose), IR treated medium ADTM (12 mg/kg) group (ADTM-M, medium dose) and IR treated high dosage ADTM (24 mg/kg) group (ADTM-H, high dose). MIR was completed after 30 min ischemia and 24 h reperfusion. ADTM was synthesized and generously provided by Dr. Yuqiang Wang (Institute of New Drugs, Jinan University, Guangzhou, China). Amlodipine (Sigma-Aldrich, St. Louis, MO, USA) is a calcium channel blocker used to treat hypertension and angina pain and is used as a positive control in the rat MIR model. ADTM and Amlodipine were administered by intravenous injection after 15 min of occlusion.

### Echocardiography and hemodynamic parameters

Echocardiography was performed on rats after MIR. An echocardiogram was performed using the ultrasound system Vevo2100 high-resolution imaging system (VisualSonics, Toronto, Ontario, Canada) and a 10 MHz imaging linear scanning probe transducer. Left ventricular ejection fraction (EF) and fraction shortening (FS) were calculated by M-mode recording method.

Hemodynamic parameters were recorded through a heparin-filled pressure sensor (from the right carotid artery to the left ventricle) docked with the BL-420N biological function experiment system (Tai Meng technology, Chengdu, China). Hemodynamic parameters include left ventricular systolic pressure (LVSP), left ventricular end-diastolic pressure (LVEDP), and maximum rate of increase and decrease of left ventricular pressure (+dp/dtmax and -dp/dtmax).

### Neonatal rat ventricular myocytes culture and transfection

The procedure for culturing neonatal rat ventricular myocytes (NRVMs) is the same as previously described 22. NRVMs were grown in Dulbecco's modified Eagle's medium (DMEM; Hyclone, Logan, UT, USA) supplemented with 10% fetal bovine serum (FBS, Hyclone) in a humidified atmosphere of 95% air-5% CO_2_ at 37 °C and subsequent experiments were performed 48 h after plating. The cells were then treated with hypoxia (1% O_2_) for 6 h and then followed by 12 h of re-oxygenation.

PAFR siRNA (20 nM) and negative control siRNA (siRNA-NC, 20 nM) were purchased from Thermo Fisher Scientific, USA. According to the manufacturer's instructions, before transfection with X-treme GENE siRNA transfection reagent (Roche, Germany), NRVMs (1 × 10^5^ per well) was starved in serum-free medium for 24 hours. Forty eight hours after transfection, NRVMs was treated with different conditions. Before PAF (20 μM, Sigma-Aldrich, USA) was added, cardiomyocytes were pretreated with ADTM (300 μM) for 15 minutes, and then exposed to HR or normal condition.

### Measurement of infarct size

After washing out remaining blood, the heart was cut into 2 mm thick slices below the ligature line and stained with 1% TTC (triphenyltetrazolium chloride, Sigma-Aldrich) at 37 °C for 15 minutes. The infarct area is stainless and the living area is red. Image ProPlus 5.0 software (Media Cybernetics, Wokingham, UK) was used to calculate the area of cardiac infarction.

### Cell viability assay

The cells were treated as designated in 96 plates, and were then incubated with 20 µl MTT (0.5 mg/ml) for 4 h. The medium was carefully removed and 200 µl DMSO was added to each well, and were rocked for 10 min. The absorbance values were detected at 490 nm using an Infinite M200 microplate spectrophotometer (Tecan, Salzburg, Austria).

### Measurement of Inflammatory Parameters by ELISA

The levels of interleukin-1β (IL-1β) and tumor necrosis factor-α (TNF-α) in heart tissues and NRVMs were determined using ELISA kits, and the detection operations were performed according to the manufacturer's instructions (R&D Systems, USA).

### PAF measurement

The PAF production were measured for heart tissue and cells by double antibody sandwich method following the manufacturer's instructions (Bioswamp Biotechnology, China).

### TUNEL staining

According to the manufacturer's instructions, the apoptosis of NRVMs and the left ventricle was detected using Terminal deoxynucleotidyl transferase dUTP nick end labeling (TUNEL) fluorescence FITC kit (Roche, USA). After TUNEL staining, the nuclear was stained using DAPI (1:100, Beyotime Biotechnology, China). Fluorescence staining was observed with a Confocal Laser Scanning Microscope (FV1000, Olympus, Japan). The apoptotic rate was calculated as TUNEL-positive cells in each field.

### Caspase-3 and LDH activity assay

Caspase-3 activity kit (Beyotime Institute of Biotechnology, Jiangsu, China) and serum LDH activity kit (Jiancheng Bioengineering Institute, Nanjing, China) were used to determine caspase-3 and LDH activity, as described in the previous study 22.

### Western blot

Total protein was extracted from NRVMs and the left ventricle as described in previous study 22. Briefly, proteins were separated by electrophoresis on SDS-polyacrylamide gels and transferred moist to nitrocellulose filter membranes. Membranes were incubated with anti-PAFR (1:200, Rabbit polyclonal, 39 kDa; Abcam, MA, USA) antibody overnight at 4 °C with the following secondary antibodies for 1 h at room temperature in the dark next day. The images were captured by the Odyssey CLx Infrared Imaging System (LI-COR Biosciences, Lincoln, NE, USA). Anti-β-actin (1:500, Rabbit polyclonal, 43 kDa; Santa Cruz, CA, USA) antibody was an internal control.

### Data analysis

The data are represented by means ± SEM. The statistical analyses were used by one-way ANOVA (for groups of ≥ 3) and Student's test (for 2 groups). In all cases, P < 0.05 was considered to be statistically significant. The data were analyzed using GraphPad Prism 5.0.

## Results

### Effect of ADTM on the cardiac function after MIR injury in rats

To study the effect of ADTM on MIR *in vivo*, rats were ligated with LAD for 30 minutes and then re-perfused for 24 hours. Left ventricular function was evaluated by hemodynamic parameters and echocardiography (Table 1). The MIR injury was confirmed by a decreased recovery of haemodynamic function. The markedly reduced LVSP, +dp/dt max and -dp/dt max, and increased LVEDP were clearly observed in the MIR group, relative to those in sham group, further signs of left ventricular dysfunction. However, the protective effect of treatment with ADTM (24 mg/kg) or Amlodipine were shown by the restoration of the post-reperfusion haemodynamic parameters (Table 1).

At the same time, compared with the sham group, heart function reduced 24 h after MIR, which was proved by echocardiographic examination showing that EF and FS were significantly reduced (Table 1). However, pre-treatment with ADTM (24 mg/kg) or Amlodipine significantly improved both EF and FS in MIR rats (Table 1). All these results clearly demonstrated the cardio-protective effect of ADTM on MIR injury *in vivo*.

### The protective effect of ADTM on rat MIR injury

First, the infarct size of individual rat hearts was evaluated by TTC staining assay (Fig. 1B). Compared with the sham group, myocardial infarction size was increased in MIR rats. However, pretreatment with ADTM (24 mg/kg) or Amlodipine significantly reduced the infarct size, compared with the IR group (Fig. 1C). ADTM (24 mg/kg) had a good protective effect on the recovery of MIR damage, so it was used for subsequent experiments. Furthermore, a sensitive index of the myocardial enzyme (LDH) in serum was measured at 24 h post-reperfusion injury (Fig. 1D). Compared with the sham group, the serum LDH activity was significantly increased in the IR group. These values were significantly reduced in the MIR rats that had been pre-treated with ADTM (Fig. 1D).

### ADTM inhibited apoptosis and inflammation in IR myocardium

**Figure 2:**
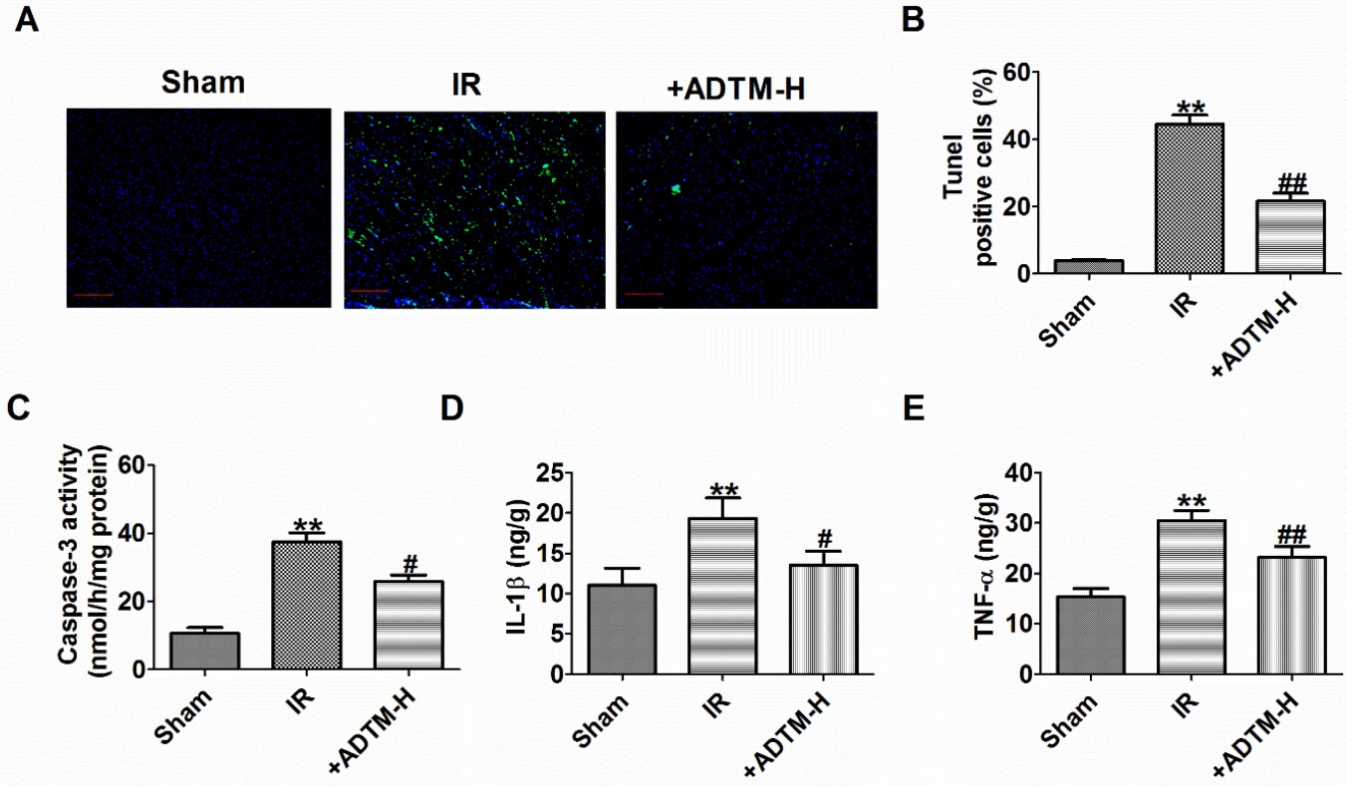
**ADTM inhibited apoptosis and inflammation in IR myocardium.** (A) Cardiac apoptosis were evaluated by TUNEL staining (nucleus stained in blue with DAPI and apoptotic cells stained in green). (B) The percentage of TUNEL-positive cell in different groups (100X). Scale bar = 100 µm. (C) Caspase-3 activity. (D) IL-1β level in hearts. (E) TNF-α level in hearts. **P < 0.01 versus Sham; ^#^P < 0.05, ^##^P < 0.01 versus IR. n = 4.

First, compared with sham rats, TUNEL positive cells were increased in myocardium of MIR rats (Fig. 2A, B). However, ADTM (24 mg/kg) significantly inhibited cardiomyocytes apoptosis in MIR rats (Figure 2A, B). The ability of mitochondria to induce apoptosis by activating downstream caspase-3, which is the main apoptotic effector molecule in the intrinsic and extrinsic apoptotic pathways 23. Therefore, we evaluated the caspase-3 activity to verify the effect of ADTM on apoptosis. The activities of caspase-3 induced by IR were markedly down-regulated by ADTM (Fig. 2C).

When MIR injury occurs, the expression of pro-inflammatory factors increases, which is mainly due to the increased release of IL-1β and TNF-α 4, 24.In order to verify the effect of ADTM on the inflammatory responses following IR injury, the myocardium levels of pro-inflammatory cytokines, including IL-1β and TNF-α, were determined in rats. As shown in Figure 2D and E, treatment with ADTM (24 mg/kg) significantly reduced the levels of IL-1β and TNF-α as compared with the IR group, indicating that treatment with ADTM might confer its cardio-protective effect by reducing pro-inflammatory cytokines in MIR injury. Together, the results show that ADTM can inhibit cell apoptosis and inflammation of MIR injury.

### ADTM regulates PAF/PAFR in MIR *in vivo* and *in vitro*

**Figure 3:**
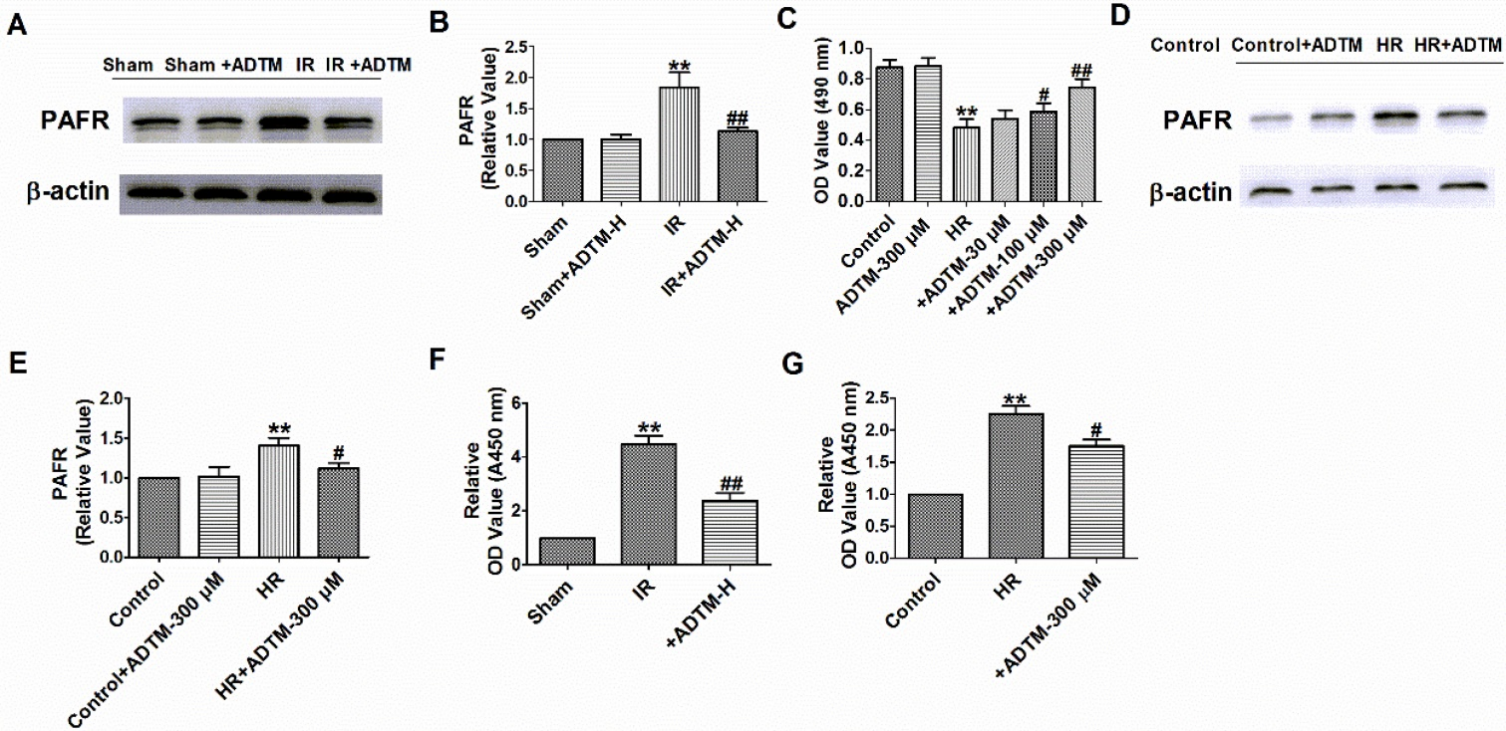
** PAFR is mediated by ADTM in MIR *in vivo* and *in vitro*.** (A&B) The relative protein level of PAFR in heart (n = 3). (C) MTT assay (n = 6). (D&E) The relative protein level of PAFR in cardiomyocytes (n = 3). (F&G) The relative level of PAF in MIR *in vivo* and *in vitro* (n = 4). B: **P < 0.01 versus Sham, ^##^P < 0.01 versus IR; C: **P < 0.01 versus Control;^ #^P < 0.05,^ ##^P < 0.01 versus HR; E: **P < 0.01 versus Control; ^#^P < 0.05 versus HR_;_ F: **P < 0.01 versus Sham, ^##^P < 0.01 versus IR; G: **P < 0.01 versus Control;^ #^P < 0.05 versus HR.

Platelet activating factor receptor (PAFR) is a G protein-coupled receptor expressed on the cell and nuclear membranes of various cell types, including epithelial and endothelial cells, macrophages and neutrophils 25, 26. Platelet-activating factor (PAF) is a potent inflammatory mediator that exerts its actions via the single PAFR and PAFR-deficiency alleviates MIR injury in mice 27, 28. Additionally, ADTM displayed cardio-protective effects in animal myocardial infarction and promoted angiogenesis served as a Ca^2+^ current blocker 18, 19 and PAF treatment increased intracellular Ca^2+^ level in isolated myocytes of guinea pig and rat ventricle, or H9c2 cells via its receptor 29-31. Therefore, we speculate that ADTM may prevent MIR possibly through PAF/PAFR regulation. PAFR was increased in MIR* in vivo*, which were reversed by ADTM (Fig. 3A-B). It is worth noting that there were no significant abnormalities in PAFR expression with ADTM treatment in sham rats (Fig. 3A-B). HR reduced cardiomyocytes viability, but it was significantly increased by ADTM (100 μM and 300 μM), and markedly more increased after HR treatment at 300 μM (Fig. 3C). Accordingly, 300 μM ADTM treated for HR was chosen for subsequent experiments. Next, we saw similar results of PAFR expression in MIR *in vitro* as *in vivo* by ADTM (Fig. 3D-E). There were no significant abnormalities in PAFR expression with ADTM treatment in normal cardiomyocytes (Fig.3 D-E). Several experiments investigating the production of PAF in the MIR heart suggest that PAF plays a relevant role in these pathophysiological conditions 32. We found that PAF production was increased in MIR *in vivo* and *in vitro*, which were reversed by ADTM (Fig. 3F-G). These results indicated that ADTM can mediate PAF/PAFR in cardiomyocytes during IR/HR.

### PAF/PAFR is critical for ADTM attenuate cardiomyocytes apoptosis and inflammation during hypoxia/re-oxygenation

**Figure 4:**
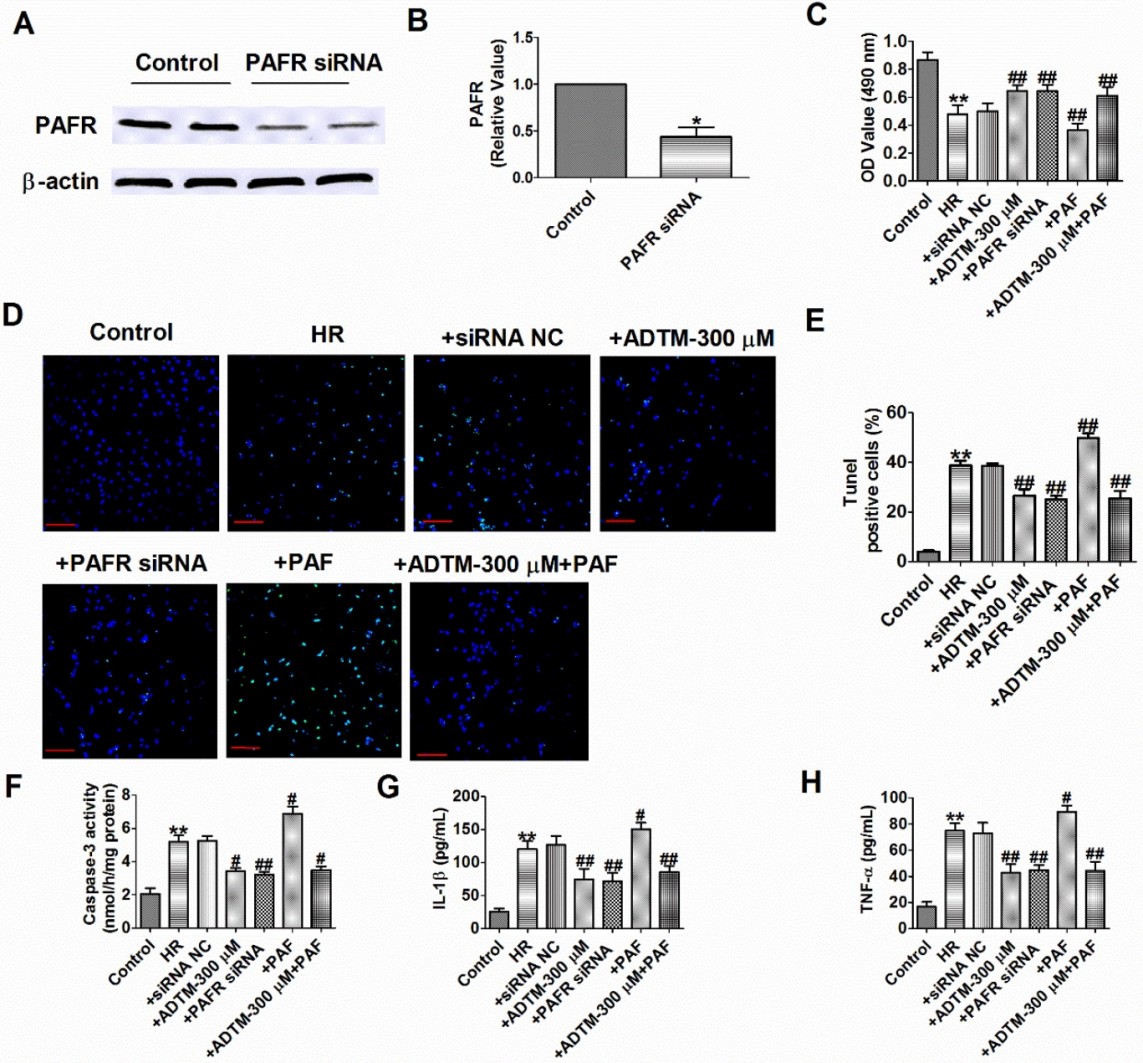
**ADTM inhibits cardiomyocytes apoptosis and inflammation by PAFR down-regulation during hypoxia/re-oxygenation.** (A&B) The relative protein level of PAFR (n = 3). (C) MTT assay (n = 6). (D) Representative images of TUNEL staining of cardiomyocytes showing the apoptotic cells. (E) Statistical results of TUNEL-positive cells per field (n = 4, 100X). Scale bar = 100 µm. (F) Caspase-3 activity (n = 4). (G) IL-1β level in cardiomyocytes (n = 4). (H) TNF-α level in cardiomyocytes (n = 4). *P < 0.05, **P < 0.01 versus Control;^ #^P < 0.05,^ ##^P < 0.01 versus HR.

Pretreatment with ADTM significantly attenuated tert-butylhydroperoxide- induced apoptosis in H9c2 cells and 6-Hydroxydopamine-induced neurotoxicity as well as production of reactive oxygen species and inflammation in PC12 cells 17, 19. PAF could induce apoptosis on the rat H9c2 cardiomyocytes and PAFR-deletion alleviates apoptosis, inflammation and oxidative stress in the mice hearts after MIR 28, 31. We subsequently aimed to evaluate the effects of ADTM, PAF or PAFR siRNA on cardiomyocytes apoptosis and inflammation during HR. First, we found PAFR protein level was significantly decreased in PAFR siRNA group (Fig. 4A, B). Compared to normal cardiomyocytes, HR reduced cardiomyocytes viability, increased the number of TUNEL-positive cells, caspase-3 activity, and the release of inflammatory cytokines (IL-1β and TNF-α) (Fig. 4C-H). However, ADTM or PAFR siRNA increased the cell viability, decreased the number of TUNEL-positive cells, caspase-3 activity and inflammatory cytokines (IL-1β and TNF-α) release in HR induced cardiomyocytes, while PAF showed the opposite effect in cell apoptosis and inflammation (Fig. 4C-H).

We further examined signaling mechanism in which ADTM mediated PAFR in HR induced cardiomyocytes. The results showed that ADTM increased the cell viability, inhibited cell apoptosis and inflammation after PAF used in HR induced cardiomyocytes (Fig. 4C-H). These data implied that ADTM attenuated HR induced cardiomyocytes apoptosis and inflammation by PAF/PAFR signaling.

## Discussion

The main findings of this study are: (1) ADTM reduces the infarct size and improves cardiac function in MIR rats; (2) ADTM inhibits myocardial cell apoptosis and inflammation in ischemia/reperfusion myocardium; (3) ADTM inhibits cardiomyocytes apoptosis and inflammation in hypoxia/re-oxygenation-induced cardiomyocytes by down-regulation of PAFR. These findings suggest that ADTM may be beneficial for therapy of MIR.

Previous studies have reported that ADTM is associated with the progression of myocardial infarction 18-20. ADTM induces vasodilation on rat aorta and exerts cardio-protection in dogs of myocardial infarction 18. After experimental myocardial infarction in mice, ADTM promotes angiogenesis and cardiac healing 19. ADTM possesses effective cardio-protective properties against oxidative injury in rat myocardium infarction 20. However, the potential role of ADTM in MIR remains unclear. As expected, we found ADTM has a significant anti-MIR damage effect *in vivo* model. We found that treatment with ADTM significantly improved the recovery of IR-induced myocardial dysfunction as improved the changes in LVSP, LVEDP and ±dP/dt max, and an increase of FS and EF, and reduce the infarct size caused by IR. We also observed that Amlodipine used as a positive control has the same protective effects as ADTM. Meanwhile, we evaluated the levels of biochemical marker of myocardial damage, such as LDH, that have been used for the diagnosis of acute myocardial infarction in clinical practice 33. The results show that ADTM can significantly reverse the IR-induced an increase of LDH activity in serum.

MIR can cause local myocardial inflammation, leading to apoptosis of myocardial cells 8. It has recently been reported that some herbs are involved in many heart pathophysiological processes (inflammation and cardiomyocytes apoptosis) of MIR 8, 34. Rosmarinic acid and oligomeric adrenal Prime 1 protects against MIR by regulating inflammation and cardiomyocytes apoptosis in animal models 8, 34. As expected, ADTM significantly increased cardiomyocytes viability, suppressed cardiomyocytes apoptosis and caspase-3 activity, and reduced IL-1β and TNF-α level in IR/HR-induced cardiomyocytes.

A recent study showed that PAFR regulates lung inflammation caused by colitis through NLRP3 signaling 35. Meanwhile, inhibiting PAFR alleviate MIR injury via reducing inflammation, oxidative stress and apoptosis 28. However, little is known regarding the role of PAFR activation or inhibition in HR-induced cardiomyocytes. In the present study, we found that knockdown of PAFR significantly decreased cell apoptosis, caspase-3 activity and inflammatory mediators (IL-1β and TNF-α) production induced by HR. In contrast, PAF causes more severe apoptosis and inflammation in HR-induced cardiomyocytes.

ADTM inhibited Ca^2+^ concentration in vascular smooth muscle cells and PAF increases Ca^2+^ concentration in H9c2 cardiomyocytes 19, 30. We therefore presume that ADTM attenuates MIR injury via regulating PAFR signaling. The results showed that ADTM down-regulated PAFR protein expression in cardiomyocytes during IR/HR. Notably, ADTM does not mediate PAFR mRNA and protein expression in normal cardiomyocytes *in vivo* and *in vitro*. As expected, PAF promoted cell apoptosis and inflammation which were reversed by co-treatment of ADTM in HR induced cardiomyocytes, and the negatively regulatory effects of ADTM on PAF production in cardiomyocytes during IR/HR. The results suggest that ADTM can improve cardiac function and reduces MIR via blocking the signaling pathway of PAF/PAFR.

Previous study showed that ADTM displayed cardio-protective effects partly through Akt/PI3K and Nrf2 pathways 20. PAFR-knockout reduced inflammation by suppressing NF-κB activation and inhibited apoptosis via JAK1/STAT1 pathway 28. Further investigations will be required to understand the other signal pathways driven by ADTM, and to explore the mechanisms that underlie inflammation and apoptosis in MIR. Taken together, these results suggest that ADTM plays a beneficial role in the pathogenesis of MIR injury by attenuating PAFR, suggesting that ADTM may be a potential and effective therapeutic agentto treat MIR. Danshensu and ligustrazine hydrochloride injection mixture is widely used in clinical to treat cardiovascular diseases in China 36. So ADTM is also expected to be used in clinical practice in the future.

## Figures and Tables

**Table 1:** Effects of ADTM on hemodynamics and heart function on IR rat

Group	Dose (mg/kg)	LVSP (mmHg)	LVEDP (mmHg)	+dp/dtmax (mmHg/ms)	-dp/dtmax (mmHg/ms)	EF (%)	FS (%)
Sham	-	149.7±3.39	1.94±0.17	5.75±0.29	5.19±0.27	82.07±2.95	57.53±2.90
ADTM	24	148.4 ± 2.54	1.97± 0.16	5.62± 0.34	5.25± 0.27	82.25±3.05	58.97± 3.65
IR	-	103.5±3.24**	5.28±0.28**	3.75±0.20**	3.44±0.23**	51.17±3.24**	25.25±1.69**
IR+ADTM	6	107.6 ± 3.82	5.33 ±0.29	3.83±0.23	3.43±0.26	51.45±2.17	25.22±2.10
	12	111.8± 4.34	5.13± 0.24	3.99± 0.25	3.62± 0.19	54.48± 2.81	26.97±1.63
	24	131.4± 3.21^##^	3.92 ± 0.25^##^	5.27 ± 0.27^##^	4.73 ± 0.22^##^	67.40 ± 3.61^#^	39.17 ±2.81^##^
I/R+ Amlod	2	133.7± 3.97^##^	3.88 ± 0.30^##^	5.35 ± 0.33^##^	4.80 ± 0.25^##^	66.43 ± 3.69^#^	38.38± 2.38^#^

Amlod, Amlodipine; LVSP, left ventricular systolic pressure; LVEDP, left ventricular end-diastolic pressure; +dp/dtmax, maximum rate of increase of left ventricular pressure; -dp/dtmax, maximum rate of decrease of left ventricular pressure; EF, ejection fraction; FS, left ventricular fraction shortening. **p < 0.01 versus Sham;^ #^p < 0.05,^ ##^p < 0.01 versus IR. n = 6.
